# The Sodium Channel B4-Subunits are Dysregulated in Temporal Lobe Epilepsy Drug-Resistant Patients

**DOI:** 10.3390/ijms21082955

**Published:** 2020-04-22

**Authors:** Mariam A. Sheilabi, Louise Y. Takeshita, Edward J. Sims, Francesco Falciani, Alessandra P. Princivalle

**Affiliations:** 1 Biomolecular Sciences Research Centre, Sheffield Hallam University, Howard Street, Sheffield S1 1WB, UK; mariam.sheilabi@gmail.com (M.A.S.); edsims93@gmail.com (E.J.S.); 2 Institute of Integrative Biology, University of Liverpool, Biosciences Building, Liverpool L69 7ZB, UK; Louise.Takeshita@liverpool.ac.uk (L.Y.T.); F.Falciani@liverpool.ac.uk (F.F.)

**Keywords:** temporal lobe epilepsy, hippocampal sclerosis, antiepileptic drug resistance, *SCN4B*, voltage-gated sodium channels, Nav β4 subunit

## Abstract

Temporal lobe epilepsy (TLE) is the most common type of partial epilepsy referred for surgery due to antiepileptic drug (AED) resistance. A common molecular target for many of these drugs is the voltage-gated sodium channel (VGSC). The VGSC consists of four domains of pore-forming α-subunits and two auxiliary β-subunits, several of which have been well studied in epileptic conditions. However, despite the β4-subunits’ role having been reported in some neurological conditions, there is little research investigating its potential significance in epilepsy. Therefore, the purpose of this work was to assess the role of SCN4β in epilepsy by using a combination of molecular and bioinformatics approaches. We first demonstrated that there was a reduction in the relative expression of *SCN4B* in the drug-resistant TLE patients compared to non-epileptic control specimens, both at the mRNA and protein levels. By analyzing a co-expression network in the neighborhood of *SCN4B* we then discovered a linkage between the expression of this gene and K^+^ channels activated by Ca^2+^, or K^+^ two-pore domain channels. Our approach also inferred several potential effector functions linked to variation in the expression of *SCN4B*. These observations support the hypothesis that *SCN4B* is a key factor in AED-resistant TLE, which could help direct both the drug selection of TLE treatments and the development of future AEDs.

## 1. Introduction

Temporal lobe epilepsy (TLE) is one of the most common causes of epilepsy in adults and can arise sporadically or with a clear familial recurrence [1]. The most common cause of TLE is hippocampal sclerosis (HS), accounting for ~80% of TLE patients and ~30.5%–45% of all epileptic cases. Other causes of TLE include benign tumors, vascular malformations, cortical development malformation and infectious gliosis [1].

The initial approach to the treatment of TLE relies on the administration of antiepileptic drugs (AEDs); about 60% of TLE patients are responsive to pharmaco-therapies. The remaining 40% are not responsive to AEDs, despite the use of 2–3 different drug regimens, including combination therapy: therefore, surgical approaches are considered [2].

In about 70% of cases, surgical resection successfully eliminates or significantly reduces seizure occurrence. One study found that 14% of the patients following temporal lobe surgery achieved long-term AED discontinuation, 50% achieved monotherapy and 33% remained on polytherapy [3]. However, this treatment is very invasive and has several undesirable side-effects, given the temporal lobes’ role in processing sensory input [4,5]. Furthermore, considering that surgery is not always successful, it is important to understand why AED resistance occurs in these individuals [6].

Despite our understanding of the binding of many AEDs with their targets, little is known about how the variable composition of the channels affects both the molecular interaction of AEDs with their targets and their pharmacological effects. The voltage-gated sodium channels (VGSCs) are perhaps the most common target for early AEDs and still are mostly used [7].

VGSCs are heteromeric complexes composed of α- and β-subunits. Each α-subunit is associated with one or more of the four β-subunits, subtypes β1, β2, β3 and β4 [8], which are homologous to cell adhesion molecules belonging to the Ig superfamily [9]. The β1 and β3 interact with the α-subunit non-covalently, whereas β2 and β4 associate via disulfide bonds [10].

The mechanism of AED resistance is most probably variable and multifactorial, depending on the cause along with the drug target [11]. There are several hypotheses to clarify the mechanism for AED resistance: (I) the multidrug transporter hypothesis shows contradicting evidence [12,13,14]; (II) the drug targets are missing, assuming a loss of target sensitivity as being the cause of the AED resistance [15,16]; (III) there is an alteration of drug targets due to polymorphism and transcriptional changes in VGSC α-subunit previously associated with epilepsy and AED resistance [17,18]. None of these theories have been fully explained or demonstrated.

The roles of the α-subunits are also well recognized in many channelopathies [19], including epilepsy [7,20]. Mutations in β1 [21] were associated with AED resistance in animal models, as are some β-subunits [8], such as *SCN2B, SCN3B* [22] and *SCN4B.* Mutations in *SCN4B* have been reported in neuropathologies linked with epilepsy [8], such as sudden unexpected death in epilepsy (SUDEP) and Dravet syndrome [8].

The physiological and pathological significance of β4, which may alter the effects of AEDs targeting VSCGs, is due to its ability in mediating the fast resurgent sodium current in the cell bodies of many neurons [23], which in fact helps in determining multiple aspects of VGSC gating [24]. The β4-subunit has been demonstrated to be involved in long-QT syndrome, atrial fibrillation and neuritic degeneration in patients with Huntington’s disease [25,26,27], as have the other β-subunits [8,22].

The VGSC β4-subunit is yet to be investigated in human TLE samples, though it has been demonstrated to be involved in a range of neurological pathologies and in determining the gating of the channel itself. Hence, the hypothesis was that *SCN4B* could be impaired in temporal lobe epilepsy with hippocampal sclerosis (TLE-HS). Therefore, we have investigated the expression modifications in the transcript *SCN4B* and the VGSC β4-subunit, the Nav β4 protein, in TLE-HS. We have identified genes co-expressed with *SCN4B* in hippocampus of TLE patients from a publicly available dataset. Functional investigation of the co-expressed genes revealed links to several pathways and components that have been previously associated to epilepsy, including potassium channels, calcium binding, mitochondria, metabolism, response to stress and methylation. Such pathways may play important roles in the pathophysiology associated with the lack of response to sodium channel blocker AEDs.

## 2. Results

### 2.1. SCN4B Transcripts Expression in HS and NC Samples

**Figure 1:**
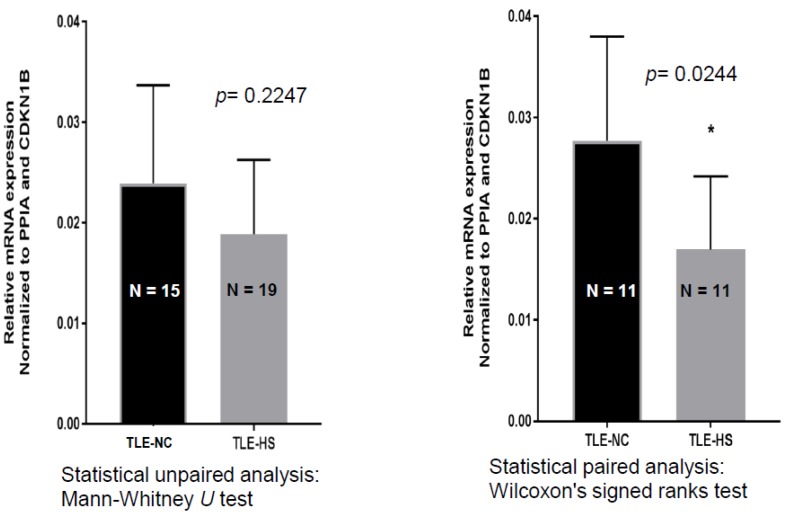
Relative expression level (2^−ΔCt^) of SCN4B gene, determine using RT-qPCR, in hippocampal sclerotic and non-sclerotic tissue samples of each patient. Left panel: unpaired samples. Right panel: paired samples. Gene expression levels were calculated using the Ct values of each sample and normalized to the house-keeping genes (PPIA and CDKN). Data are means ± SD, where *n* = 3 for control and epileptic samples of each patient.

In order to test the hypothesis that *SCN4B* is differentially expressed in hippocampus of pharmaco-resistant TLE-HS patients and adjacent (non-epileptic) neocortex tissue (designated as temporal lobe epilepsy neocortex (TLE-NC)) [28], real-time qPCR analysis was used, and *SCN4B* expression was normalized to the house-keeping genes (HKGs). The results from comparing *SCN4B* expression in TLE-NC and TLE-HS, by using unpaired analysis, show a decreased expression of *SCN4B*; however, this was not statistically significant (*p* = 0.2247) (Figure 1). When comparing TLE-HS and TLE-NC across the 11 patient samples, with a paired analysis, *SCN4B* downregulation in TLE-HS is statistically significant, with a 43% decrease in expression (*p* = 0.0244).

### 2.2. Western Blot Analysis of Nav β4 in HS and NC Samples

**Figure 2:**
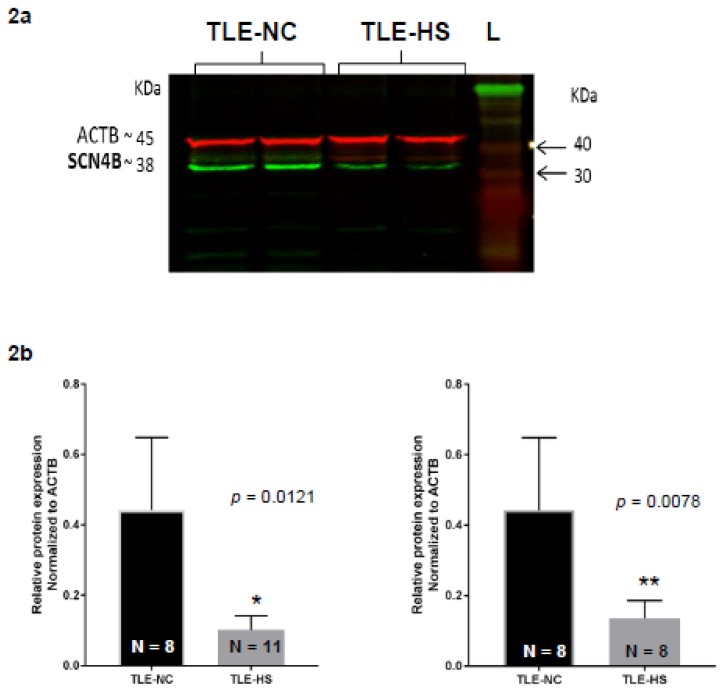
Qualitative example and quantitative Western blot analysis of β-actin and Nav β4 protein expression in sclerotic and non-sclerotic samples of patients with AED-resistant TLE. (**a**) A Western blot analysis of Nav β4 (37 kDa) and β-actin (45 kDa) in sclerotic and non-sclerotic hippocampal tissue. Protein extracts were run on SDS–polyacrylamide gels and analyzed by Western blotting. TLE-NC, non-sclerotic tissue; TLE-HS, hippocampal sclerotic tissue; L, Chameleon duo prestained protein ladder tissue. (**b**) Relative Nav β4 protein expression level, compared to the β-actin control, as determine by densitometry. Data are means ± SD, where *n* = 8 for TLE-HS patients and *n* = 11 for TLE-NC controls.

It is important to determine whether the transcriptional changes determined by qPCR are reflected in the protein levels of the *SCN4B* gene product Nav β4. Therefore, protein extracts from the hippocampal non-sclerotic and sclerotic tissue of each patient were subjected to Western blot analysis, staining for Nav β4 (Figure 2a). The normalized band densities were compared between the non-sclerotic and sclerotic tissue proteins for each patient to determine the relative protein changes in Nav β4 (Figure 2b). A reduction in the relative protein expression levels was observed.in sclerotic hippocampal tissue compared to non-sclerotic tissue resection. In this case, we used both unpaired and paired analysis, and the decrease in TLE-HS was statistically significant for both types of analysis (*p* = 0.0121 and *p* = 0.0078 for unpaired and paired, respectively).

### 2.3. SCN4B Co-Expression Network Analysis in TLE Patients

After demonstrating that sclerotic hippocampus tissue in drug-resistant TLE patients is characterized by lower expression of *SCN4B*, both at the mRNA and protein level, we investigated the functional implications of this variability in *SCN4B* expression. This challenge was addressed by generating a co-expression network using a publicly available transcriptomics dataset from TLE hippocampus samples, followed by the investigation of functional aspects of genes significantly co-expressed with *SCN4B*. A total of 83 genes were found significantly co-expressed with *SCN4B* (i.e., FDR < 0.05 and posterior probability > 0.8), but only 77 genes contained functional information available.

**Figure 3:**
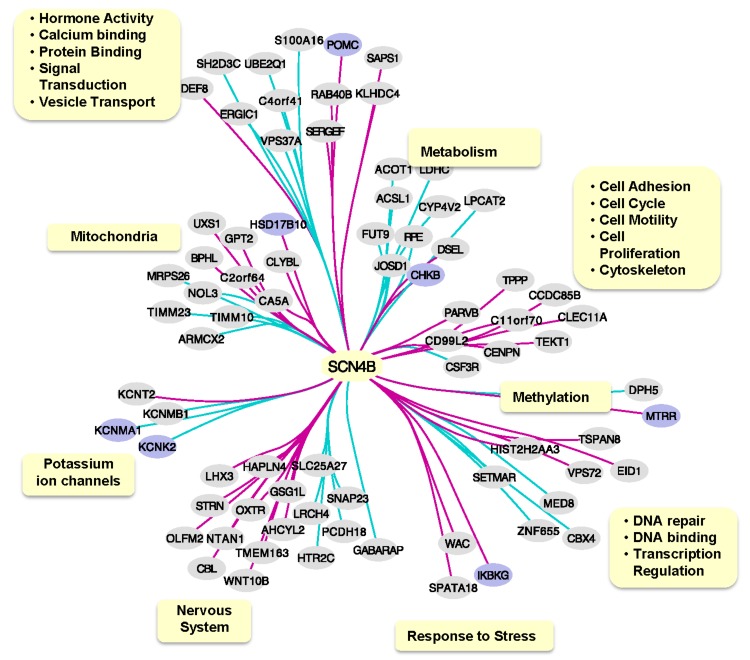
Scheme summarizing 77 genes co-expressed with SCN4B (FDR < 0.05) and their functional annotation. An overview of their functional groups is presented on yellow boxes, based on associated gene ontology terms. Blue nodes indicate genes previously associated to epilepsy according to DisGeNET database. Green edges indicate negative correlation, while magenta edges indicate positive correlation. Blue lines: positive co-expression. Purple lines: negative co-expression.

Using information retrieved from both DAVID and AmiGO 2, we have annotated relevant GO terms associated to co-expressed genes and have identified several biological mechanisms and components associated to these genes, including components closely related to neuronal function such as potassium ion channels (Figure 3). Additionally, seven of the co-expressed genes were previously found associated to epilepsy, which seems to be associated to a diversity of biological mechanisms (hormone-based signaling, mitochondria, metabolism, potassium ion channels, methylation and response to stress). DAVID analysis found no enriched pathways for *SCN4B* co-expressed genes. In contrast, STRING analysis found that calcium-activated potassium channel activity GO term (GO:0015269) was significantly enriched (FDR = 0.0374) due to *KCNMA1*, *KCNMB1* and *KCNT2* genes.

## 3. Discussion

In this study, patients affected by mesial TLE were compared with an adjacent specimen from non-seizing medial temporal neocortex tissue, mesial TLE-NC. The experiments demonstrate a reduction in the expression profile, at a transcriptional and translational level, in the voltage-gated sodium channel β4-subunit in the sclerotic hippocampal tissue of AED-resistant TLE compared with TLE-NC. Changes in mRNA expression levels are not always reflected in the protein expression, but our results demonstrate a borderline correlation. This evidence potentially elucidates a key factor of AED resistance. The β4-subunit plays an important role in determining the gating of the VGSC and can therefore affect the binding sensitivities of AED, many of which act in a use-dependent manner.

A reduction in the Nav β4 could simply result in a loss of drug action on the target channel, contributing to AED resistance in TLE patients. However, the β4-subunit has already been shown in cell models to favor the open state of VGSCs and promote persistent currents with certain α-subunits [29]. Therefore, a reduction is more likely to result in a decrease in the frequency and duration of VGSC in the resting state, which would reduce the opportunity for the vast number of use-dependent VGSC-modulating AEDs to bind to their target.

The combination of β-subunits defines a range of VGSC current amplitudes. Co-expression of β1 with β4 can in fact suppress the gating effects of β4 mentioned previously. This is a result of β1-subunits’ own effect on VGSC gating, which instead stabilizes the closed states at hyperpolarized potentials and inactivated states at depolarized potentials [29]. Therefore, a pathology, like TLE-HS, that results in a reduction of β4 in an area of the brain that would usually express β4, would in fact have an enhanced effect. There would be a loss in the favor of the open/resting state of VGSCs caused by β4 association and an increase in the effects of β1 on the VGSC gating, which are usually partially suppressed through co-expression with β4. In turn, these increased effects of β1 would further reduce the binding of AEDs to VGSCs.

Despite understanding the possible influence that a reduction in β4 may have on AED resistance, it is important to identify the cause of this lower expression to help discover new molecular targets to manipulate β4 expression. These experiments do not allow us to determine why these changes occur. They could be a result of epilepsy or the sclerosis. Other types of epilepsy, with and without sclerotic tissue, and other pathologies associated with the formation of sclerotic tissue, like multiple sclerosis (MS), would need to be analyzed in order to develop our understanding of a potential cause for this reduction in β4.

Another approach that could help further direct this research is the determination of whether there are mutations in the *SCN4B* promoter site in TLE-HS patients. Methylation and acetylation patterns should also be investigated, as these may result in altered β4 expression levels, but they are yet to be investigated in any of the β-subunits. Nevertheless, increased expression patterns of DNA methyltransferase (*DNMT*) isoforms *DNMT1* and *DNMT3* [30] and histone deacetylases (*HDAC2*) [31] have been found in our analysis in TLE. Our data also show two more genes co-expressed with *SCN4B*: methionine synthase reductase (*MTRR*) and diphthine synthase (*DPH5*). They are both involved with the methylation process in neurons, and they show opposite co-expression with *SCN4B*, *MTRR* with a positive and *DPH5* with a negative co-expression. *MTRR* was already investigated in epilepsy, [32], whereas *DPH5* has been linked to the translation elongation factor 2 but has not been investigated yet in TLE, and this could lead to significant modulation of the translational regulatory machinery.

Mutations in other β-subunits have already been found. For example, heterozygous mutations in extracellular domain of *SCN1B* [33] and several additional mutations have all been associated with epilepsy [33,34,35,36,37]. Therefore, it appears reasonable to hypothesize that mutations affecting the structure and expression of β4 may be present in epilepsy, including TLE.

The diffused expression of Nav1.2 along some demyelinated axons supports recovery of action potential conduction. Polymorphisms in the *SCN2A*, which may reduce this recovery action, have been associated with epilepsy and AED resistance [18]. In contrast, Nav1.6 produces Na^+^ current that can drive the Na^+^–Ca^2+^ exchanger, potentially triggering injurious cascades and promoting neuronal atrophy [38]. Seizures also result in an increase in Na^+^ currents, which also drives neuronal atrophy: ‘seizure-induced neuronal atrophy’ [39]. Therefore, genetic and expressional changes in β4, along with other VGSC-subunits, in TLE could alter the gating of VGSCs and may alter the neuroprotective activity of the VGSCs in the neuronal atrophy seen in the formation of sclerosis. When considered along with the inflammatory abnormalities seen in TLE [40], this suggests that TLE drives the formation of HS in predisposed neuronal tissue.

Interestingly, the β-secretase, β-site APP-cleaving enzyme 1 (*BACE1*), has shown, in Purkinje neurons, to have a novel function in the regulation of neuronal excitability by impairing the regular processing of β4 [41,42,43]. The mechanism behind transcriptional regulation of *SCN4B* remains elusive. However, it is noted that *BACE1* is known to have some transcriptional effects. Tts involvement in proteolytic cleaving of β1 is proposed to release a soluble intracellular domain of β1 which then enhances transcription of α-subunit genes, thus demonstrating the need to investigate any effects *BACE1* may have on β4 transcription as well as protein cleavage. When the potential effects of *BACE1* in sclerosis are combined with the loss of neuroprotective activity, the effects could result in the significant reduction of β4 and, hence, a reduction in the VGSCs susceptible to AED interaction. Alternatively, it may only be one of these mechanisms that results in a reduction of β4, but they would cause a reduction nonetheless.

AED resistance is likely to be multifactorial, which was demonstrated by VGSC mutations associated with AED resistance [17,18,21] and the altered expression of receptors [44]. This research elucidates the reduced expression of β4 as an important factor in the AED resistance seen in TLE-HS and could therefore direct both our current approach to the treatment of AED-resistant TLE and the development of novel AEDs in the future. The large number of AEDs with known VGSC-modulating properties, even in the small sample cohort used in this study, Table 1, helps signify the potential impact that a change in this molecular target might actually have on the approach to treating TLE.

Our network analysis using publicly available data revealed that important cellular (neuronal and glial) functions and pathways are indeed defining the phenotype of TLE in the AED-resistant cohort. This has allowed us to determine a principal element to be further investigated in order to improve the response to sodium channel blockers as therapeutics for TLE. Despite the analysis being more focused on the β1-subunit, it has been reported that sodium β channel subunits are multifunctional and act via multiple signaling pathways. This was shown in an animal model with induced mutations and by genetic characterization derived from epileptic patients affected by Dravet syndrome (DS), generalized epilepsy with febrile seizures plus (GEFS+), sudden infant death syndrome (SIDS) and sudden unexpected death in epilepsy (SUDEP) [8].

**Figure 4:**
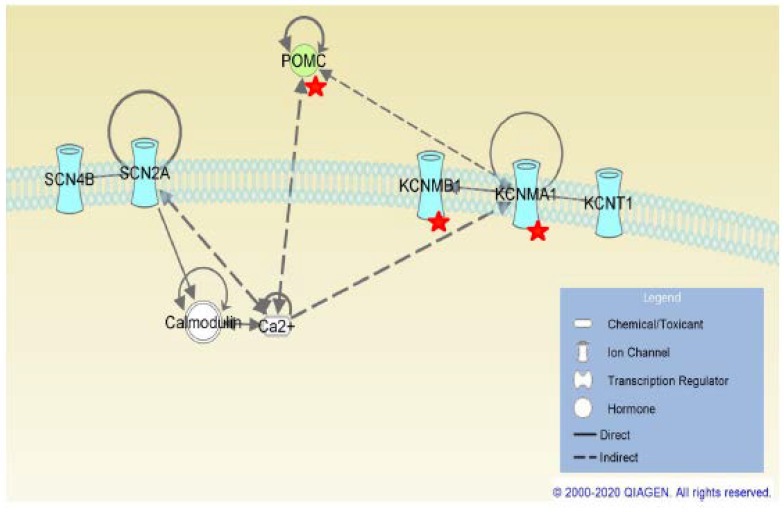
Putative mechanism involving SCN4B (IPA findings). Molecules with a red star are co-expressed with *SCN4B*. Figure produced using Ingenuity Pathway Analysis (IPA) (QIAGEN Inc., https://www.qiagenbioinformatics.com/products/ingenuity-pathway-analysis).

Differently, we have defined most, if not all, of these pathways focusing on sodium channel β4-subunit and in human brains affected by TLE (Figure 4).

The pathways that we have identified by *SCN4B* co-expression network analysis in TLE patients can be used to generate hypotheses of the mechanisms linked to impairment in the expression of *SCN4B*. These include the modulation, trafficking and localization of the sodium channel itself, the cellular migration and proliferation including the cytoskeleton, linking it to development phenomena; pathways which involve DNA regulation, repair/binding and transcription; and pathways involving protein binding and signal transduction.

Several *SCN4B* co-expressed genes are related to mitochondrial function, and its impairment has been identified as a potential cause of epileptic seizures [45]. One of these mitochondria-related genes is *CA5A* (carbonic anhydrase 5A), which has a positive co-expression with *SCN4B*. Several AEDs act through multiple mechanisms and simultaneously inhibit carbonic anhydrases and block VGSCs [44]. This example could point that other effects associated to the lower expression of *SCN4B*, in this case a correlated lower expression of *CA5A*, could simultaneously contribute to AED resistance. Mitochondrial disorders are often the cause of seizures, and diverse types of seizures have been reported to be associated with mitochondrial oxidative stress, which gradually disrupts the homeostasis of intracellular Ca^2+^. Mutations have been reported in HSD17B10, impairing the activities of the mitochondrial RNase P complex [46].

Interestingly, *SCN4B* was shown to be negatively co-expressed with two calcium-activated potassium channel genes (*KCNMA1* and *KCNMB1*) and a two-pore potassium channel (*KCNK2*) and positively co-expressed with a sodium-activated potassium channel gene (*KCNT2*).

The *KCNMA1*, *KCNMB1*, and *KCN2* genes have a main role in stopping or reducing excitatory stimuli in neurons [47]. Therefore, from these results, we can hypothesize that when *SCN4B* is dysregulated these sodium- and potassium-activated channel, they lose their inhibitory role, leading to the TLE.

There have been no previous associations of *SCN4B* with these potassium channel genes; therefore, the cause of their co-expression in TLE patients is unclear. Nevertheless, by using IPA to investigate possible pathways to link *SCN4B* to these potassium channel genes, we have found that *SCN2A*, which binds to *SCN4B* to form the sodium channel, also binds to Ca^2+^/calmodulin complexes. Mutations in *SCN2A* cause Ca^2+^ to dissociate from calmodulin, leading to higher intracellular Ca^2+^ concentration, and these changes could in turn have a downstream effect in the expression of calcium-activated potassium channels. One hypothesis is that the lower expression of *SCN4B* could also affect the ability of *SCN2A* to bind to Ca^2+^/calmodulin complexes. Interestingly, changes in *KCNMA1* expression and in intracellular Ca^2+^ concentration also have an effect on *POMC* regulation and intra/extracellular concentrations of its products. *POMC* was also found to be co-expressed with *SCN4B* in our analysis, and it has been previously associated to epilepsy. The intracellular neuropeptide alpha-melanocyte stimulating hormone (alpha-MSH) *POMC* product increases Ca^2+^ level. Depletion of extracellular Ca^2+^ decreases adrenocorticotropic hormone (*ACTH*) *POMC* product release.

There is still much to uncover about biological mechanisms in which *SCN4B* is involved and its role in AED resistance. This functional analysis of putative systems affecting or affected by *SCN4B* expression, despite the limited sample size, points to avenues that can be explored for further understanding of AED resistance and potential candidate drug targets to be considered.

## 4. Materials and Methods

### 4.1. Tissue Collection

Tissue from the hippocampal formation was obtained from pharmaco-resistant mesial TLE-HS (*n* = 19) patients and adjacent (non-epileptic) medial temporal neocortex tissue TLE-NC (*n* = 15) [28], from Sheffield Hallamshire Hospital (Table 1). Informed consent was given by all participants for experimental use; approved by South Yorkshire NRES committee York and Humber (08-H1310-49).

TLE-HS and TLE-NC sample pairs were from the same patient, therefore eliminating inter-individual, age-related, postmortem-related and drug-related variabilities.

### 4.2. qRT-PCR Analysis of SCN4B

The quantification of mRNA levels of *SCN4B* was performed by qRT-PCR in TLE-NC (*n* = 11) and TLE-HS (*n* = 19) samples. The mRNA was extracted from the epileptic patient samples and purified using the SV Total RNA Isolation System (Promega, UK), and then the product was quantified using the Nano Assay (Nanodrop Analyzer, Thermofisher, Loughborough, UK). The reverse transcription of each mRNA sample was done using 1μg of RNA with the Superscript III First-stand kit (Qiagen, UK). Gene expression levels were then determined using the TaqMan Fast Universal MasterMix (Applied Biosystem, Life Technologies, Loughborough, UK) with the StepOnePlus Real-Time PCR System (Applied Biosystems, Thermo Fisher, UK). Real-time qPCR reactions were run over 50 cycles (initial heating of 95 °C for 20 s followed by cycles of 95 °C for 1 s and 20 s at 60 °C) with 20 ng of cDNA per reaction to amplify specific genetic sequences. The following primers were used to detect specific house-keeping genes (HKGs): peptidylprolyl isomerase A (*PPIA*) (Hs04194521_s1, Applied Biosystems, Life Technologies, UK) and cyclin-dependent kinase inhibitor 1B (*CDKN1B*) (Applied Biosystems, Life Technologies, UK). These results were utilized to normalize the gene expression levels of the target gene, *SCN4B* (Hs03681025_m1, Applied Biosystems, Life Technologies, UK). All samples were run in triplicate, and only the reactions that produced appropriate amplification curves were analyzed, as produced by the real-time qPCR analytical software (StepOne Software v2.0 (Applied Biosystems, Thermo Fisher, UK)). Relative expression of SCN4β compared to an average of the HKG expression was calculated using the 2^−ΔCt^ and 2^−ΔΔCt^ methods.

### 4.3. Western Blot Analysis of Nav β4

Tissue samples were lysed using 200 mg/mL CellLytic MT Mammalian Tissue Lysis/Extraction Regent (Sigma, Welwyn Garden City, UK) and 1 mg/mL stock protease inhibitor cocktail (Sigma, UK) to extract protein. Protein concentration was determined using a bicinchoninic acid (Sigma, UK) assay. The protein samples were then subjected to electrophoresis and separated on a 10% polyacrylamide gel, using 30 µg of protein per lane, alongside the molecular weight marker, Chameleon (Life Sciences, Applied Biosystems). The gel was then blotted onto a nitrocellulose blotting membrane, Amersham Protan supported 45 µm NC (GE Healthcare, Life Technologies, Little Chalfont, UK), using 75 V (~180 mA) (PowerPac Basic, BioRad, Deeside, UK) for 50 min in transfer buffer (25 mM Tris, 193 mM glycine, 10% methanol made up with water). Then, the blot was incubated overnight in blocking solution (5% non-fat powdered milk in TBST (10 mM Tris, pH 7.5, 50 mM NaCl and 0.05% Tween-20, (Sigma)). After blocking, the blots were incubated at room temperature for 1 h in the primary antibody solution; rabbit anti-*SCN4B* (1:500) (ab80539, Abcam, Cambridge, UK) and β-actin (8H10D10) mouse mAb (1:1000) (Santa Cruz, Harrogate, UK) diluted in blocking solution. The blots were washed three times, each for 10 min, in TBST. Primary antibody binding was detected by incubation with a solution of 1:10,000 dilution of infrared IRDye (800 CW)-conjugated donkey anti-rabbit (LI-COR Biosciences, Cambridge, UK) and 1:50,000 dilution of infrared IRDye (680 CW)-conjugated donkey anti-mouse (LI-COR biosciences, Cambridge, UK) in 5% powdered milk in TBST, for 1 h at room temperature. Again, the blots were washed three times, each for 10 min, in TBST. Blots were visualized and quantified using Odyssey LI-COR Scanner (LI-COR Biosciences, Cambridge, UK).

### 4.4. Western Blot Band Quantification

The protein band densities on the Western blots, as detected by the Odyssey LI-COR scanner (LI-COR Biosciences), were quantitated using the Image Studio software (Ver3.1, Applied Biosystems). The protein bands of interest in each sample, corresponding to the Nav β4 protein (37 kDa), were normalized to β-actin (42kDa) band density, and relative intensities were calculated to run comparative analysis between the sclerotic and control (non-sclerotic) samples.

### 4.5. Statistical Analysis of SCN4B Gene Expression and Nav β4 Band Intensities

The changes in *SCN4B* gene expression and Nav β4 protein expression between sclerotic and control samples were analyzed for statistical significance using Mann–Whitney U test for the unpaired data sets. Wilcoxon’s signed ranks test was used for statistical paired analysis.

Comparative analysis, investigating correlation between the *SCN4B* and Nav β4 expression level, was performed using Spearman’s rank test to produce a correlation coefficient (strong correlation when *r* ≥ 0.7).

### 4.6. SCN4B Co-Expression Network Analysis in TLE Patients

Microarray expression profiles from 129 TLE patients (GEO ID: GSE63808) were used for inferring co-expression networks [47]. To increase the power for detecting significant correlations, probes were filtered by expression level (probes showing a detection *p*-value < 0.05 in at least 20% of the samples) and expression variation (probes having a coefficient of variation (CV) of gene expression greater than the median CV calculated across all probes in the set). After filtering and collapsing probes to gene level, a total of 7475 remaining genes were used as input for co-expression network inference using Graphic Gaussian Models (GGM) [48] method through the “GeneNet” [49] package in R (http://www.r-project.org/). This method estimates partial correlations within the probe set, identifying co-expression between probe pairs, while removing effects from other probes. A mixture model is then fitted to the partial correlations for assessing significant edges in the network.

### 4.7. Functional Analysis of SCN4B Co-Expressed Genes

We used DAVID v6.8 [50] and STRING web tools to perform functional enrichment analysis [51]. We also used Ingenuity Pathwy Analysis (IPA) (QIAGEN Inc., https://www.qiagenbioinformatics.com/products/ingenuity-pathway-analysis) [52] to investigate putative pathways linking SCN4β to co-expressed genes. IPA built-in tools, such as ‘Connect’, ‘Path Explorer’ and ‘Grow’ search for connections between selected molecules based on prior knowledge. Genes previously associated with epilepsy were retrieved from DisGeNet [53] database v6.0 (disease ID: C0014544). We also searched AmiGO 2 [54,55] database v2.5.12 for gene ontology (GO) terms associated to co-expressed genes.

## 5. Conclusions

It may be possible to specifically target the β4-subunit of sodium channels; as it only shares 35% amino acid identity with the most closely associated β2-subunit, it would be more effective to induce an overall increasing expression of β4, rather than to modulate the relatively few β4-subunits that are still present. Investigations into mutations and the transcriptional regulation of *SCN4B* would be needed to elucidate potential targets for modulating *SCN4B* expression and the pathways it influences.

## Figures and Tables

**Table 1:** Clinical data of patient samples.

Patients	Gender	Age at Surgery (years)	Duration of Epilepsy (years)	Sample Side	Current AED	Previous AEDs
Pt. 13	F	24	23	TLE-HS (Lt)	LCS, LEV	CBZ *, LMT *
Pt. 18	M	48	47	TLE-HS (Rt) TLE-NC (Rt)	PHT, LEV, LMT, GBP, TPM, PGB, ZNS	PHT *, LEV, LMT *, GBP, TPM *, PGB, ZNS
Pt. 21	M	32	3	TLE-HS (Rt) TLE-NC (Rt)	LMT, OXC	CBZ *, LEV
Pt 24	F	54	53	TLE-HS (Lt) TLE-NC (Lt)	LMT, PGB	GBP, VPA *, PHT *, CBZ *, PB, LEV, CNP, LCS
Pt 27	F	27	6	TLE-HS (Lt) TLE-NC (Lt)	NA	NA
Pt. 31	F	44	10	TLE-HS (Lt)	LCS, LEV, PHT	OXC *, LMT *
Pt. 33	M	35	14	TLE-HS (Lt)	LMT, CLB	VPA *, ZNS, CBZ *
Pt. 39	M	33	31	TLE-HS (Lt)	LEV, LMT*	CBZ*, VPA*, CLB
Pt. 41	F	39	37	TLE-HS (Rt)	None	GBP *, LEV, LMT * CBZ *, LMT *, VPA *
Pt. 46	M	48	41	TLE-HS (Rt)	None	PHT *, VGB, CLB, TPM *, TGB
Pt. 47	M	48	NA	TLE-HS (Lt)	NA	OXC *

CBZ, Carbamazepine; CNP, Clonazepam; GBP, Gabapentin; LEV, Levetiracetam; LMT, Lamotrigine; OXC, Oxcarbazepine; PB, Phenobarbital; PER, Perampanel; PGB, Pregabalin, PHT, Phenytoin; TGB, Tiagabine; TPM, Topiramate; VGB, Vigabatrin; VPA, Valproate. NA: not available. *AEDs with VGSC-modulating properties.

## Abbreviations

ATP	Adenosine triphosphate
AEDs	Antiepileptic drugs
ABC	ATP-binding cassete
BCA	Bicinchonic acid
BACE1	β-site APP-cleaving enzyme 1
DBS	Deep brain stimulation
HS	Hippocampal sclerotic
MS	Multiple sclerosis
TBS	Tris-buffered saline
TLE	Temporal lobe epilepsy
TLE-NC	Temporal lobe epilepsy neocortical
TLE-HS	Temporal lobe epilepsy hippocampal sclerotic
VGSC	Voltage-gated sodium channel
VNS	Vagus brain stimulation
